# Role of community centers in promoting sustainable regional life of community-dwelling older adults with frailty

**DOI:** 10.1093/geroni/igab046.2874

**Published:** 2021-12-17

**Authors:** Akiko Nishino, Ryogo Ogino, Takahiro Miura, Ken-ichiro YABU, Kanako TSUTSUMI, Junichiro OKATA, Kazuhiko Nishide, Tohru IFUKUBE

**Affiliations:** 1 Former The University of Tokyo, bunkyo-ku, Tokyo, Japan; 2 Saga University, Saga, Saga, Japan; 3 National Institute of Advanced Industrial Science and Technology (AIST), Kashiwa, Chiba, Japan; 4 the University of Tokyo, the University of Tokyo, Tokyo, Japan; 5 Former The University of Tokyo, Bunkyo－Ｋｕ, Tokyo, Japan; 6 The University of Tokyo, bunkyo-ku, Tokyo, Japan

## Abstract

Japan’s long-term care insurance system, which is a formal service, focuses only on older adults requiring care and support. Therefore, to create supportive communities for frail older adults, appropriate measures have been taken to establish community centers within their walking distance. However, the specific functions of these centers largely remain unknown. Accordingly, this study is aimed at clarifying the role of community centers by analyzing their services and management systems. In February 2020, we conducted a questionnaire survey (36% response rate) and four semi-structured interviews in O city, which has 36 community centers (81.45㎢, 36.4% elderly population). Results from the questionnaires revealed that the most frequent users of the community center were in their 70s (61.5%); such centers tended to provide informal services, such as exercises and cafes. Meanwhile, 57.2% of community centers collaborate with formal service providers. Community centers tend to be operated together with parent facilities, such as hospitals and nursing homes(61.2%). The results of the onsite survey showed that, in three cases, the community centers were situated within 200 meters of the parent facility. The findings show that these community centers facilitated creation of a supportive community that provides informal services to the frail elderly. Furthermore, they are operated in cooperation with formal service providers, hospitals, and nursing care facilities and are located in close proximity to one another. To summarize, the community centers continue to play a role in providing seamless services to the frail elderly even as their physical functions evolve.

